# Molecular genetic diversity and linkage disequilibrium structure of the Egyptian faba bean using Single Primer Enrichment Technology (SPET)

**DOI:** 10.1186/s12864-024-10245-x

**Published:** 2024-06-28

**Authors:** Ahmed Sallam, Ahmed Amro, Amira M. I. Mourad, Abdallah Rafeek, Andreas Boerner, Shamaseldeen Eltaher

**Affiliations:** 1 Genebank Department, Leibniz Institute of Plant Genetics and Crop Plant Research (IPK), Stadt Seeland, Germany; 2 Department of Genetics, Faculty of Agriculture, Assiut University, Assiut, 71526 Egypt; 3 Department of Botany and Microbiology, Faculty of Science, Faculty of Assiut University, Assiut, 71526 Egypt; 4 Department of Agronomy, Faculty of Agriculture, Assiut University, Assiut, 71526 Egypt; 5 Department of Plant Biotechnology, Genetic Engineering and Biotechnology Research Institute (GEBRI), University of Sadat City, Sadat City, Egypt

**Keywords:** *Vicia faba* L, Genetic diversity indices, SNP marker, STRUCTURE, Kinship, LD decay

## Abstract

Faba bean is an important legume crop. The genetic diversity among faba bean genotypes is very important for the genetic improvement of target traits. A set of 128 fab bean genotypes that are originally from Egypt were used in this study to investigate the genetic diversity and population structure. The 128 genotypes were genotyped using the Single Primer Enrichment Technology (SPET) by which a set of 6759 SNP markers were generated after filtration. The SNP markers were distributed on all chromosomes with a range extending from 822 (Chr. 6) to 1872 (Chr.1). The SNP markers had wide ranges of polymorphic information content (PIC), gene diversity (GD), and minor allele frequency. The analysis of population structure divided the Egyptian faba bean population into five subpopulations. Considerable genetic distance was found among all genotypes, ranging from 0.1 to 0.4. The highly divergent genotype was highlighted in this study and the genetic distance among genotypes ranged from 0.1 and 0.6. Moreover, the structure of linkage disequilibrium was studied, and the analysis revealed a low level of LD in the Egyptian faba bean population. A slow LD decay at the genomic and chromosomal levels was observed. Interestingly, the distribution of haplotype blocks was presented in each chromosome and the number of haplotype block ranged from 65 (Chr. 4) to 156 (Chr. 1). Migration and genetic drift are the main reasons for the low LD in the Egyptian faba bean population. The results of this study shed light on the possibility of the genetic improvement of faba bean crop in Egypt and conducting genetic association analyses to identify candidate genes associated with target traits (e.g. protein content, grain yield, etc.) in this panel.

**Supplementary Information:**

The online version contains supplementary material available at 10.1186/s12864-024-10245-x.

## Introduction

Faba bean (*Vicia faba* L.) is a significant legume crop in North and East Africa, particularly in the Arabian regions. The faba bean is one of the first domesticated crops, with cultivation dating back to the Stone Age [1]. The Middle East is frequently cited as the region of origin, although alternative research [2, 3] points to Central Asia. Egypt uses faba beans extensively since they are an affordable food with good nutritional value, especially when it comes to their high protein content [4]. The average total protein content of faba beans is 29%, and they have a high production potential. Additionally, it is one of the best nitrogen fixers and requires little to no artificial nitrogen fertilizer for optimal growth [5, 6]. Around 5.7 million tonnes of faba beans were produced worldwide in 2020, which is an increase that is nearly 55% since 2000 according to the latest FAO data https://www.fao.org/faostat/en. However, it grows in a variety of temperature zones, from boreal to subtropical and warm temperate places, where it is planted as a winter crop [5–7]. In general, faba beans grow well in the cool and moist conditions found in temperate climates. Since no wild faba bean progenitor has ever been discovered and Vicia faba does not crossbreed with other Vicia species, all the faba bean's genetic diversity has been preserved in germplasm collections and locally maintained farmer populations [1]. Genetically, faba been is an annual diploid legume (2n = 2x = 12 chromosomes) with a 13,000 Mb genome size [8], and it’s a cross-pollinated plant with a high natural outcrossing rate of 19–49%, is the only cultivated species of *Vicia L*. The high percentage of outcrossing in faba bean variants makes breeding beans extremely difficult. Numerous biotic and abiotic factors influence the outcrossing rates of faba beans, with bees being regarded as one of the key pollinators [4]. Since there are now no efficient transgenic technologies for faba beans, continuing breeding programs heavily rely on the utilization of genetic variation that already exists [5]. A better understanding of the population structure and genetic diversity in the accessible faba bean germplasm is essential for the best crop improvement. To date, numerous studies have used various molecular markers and germplasm collections to analyze the genetic relationships and diversity of faba bean germplasm ex. [5, 6, 9–14]. The genetic diversity of thirteen European small-grain faba bean accessions, six European large-grain accessions, and nine Mediterranean accessions was analysed by [15] using RAPD markers. They discovered that the genetic diversity of the small-grain accessions was rather high. Also, [16] examined the genetic variation of 22 faba bean accessions from Turkey and the ICARDA (International Centre for Agricultural Research in the Dry Areas) using 25 polymorphic SSR markers. They discovered that there was enough genetic diversity among the faba bean accessions to be useful for breeding projects. By utilizing 32 SSR markers to examine population structure diversity, [17] discovered a correlation between the geographic origins of the faba bean populations and their segregation. To improve agricultural genetic diversity, an extensive comprehension of germplasm resources' genetic diversity is essential. Because of its many genomic loci in the genome, wide distribution, genetic stability, ease of genotyping, and high-throughput computerized analysis, single-nucleotide polymorphism (SNP) has been widely utilized in the genetic diversity analysis of faba beans after the development of molecular marker technology. Such as, [18] used 768 SNP markers, assessed the genetic diversity of 45 faba bean varieties, and divided them into two main groups, the second of which can be further split into three subgroups. Recently [19], used the 130K targeted next-generation sequencing (TNGS) genotyping platform to genotype 410 worldwide faba bean accessions, yielding a total of 38,111 high-quality SNP loci through high-standard filtering and they found 410 accessions were divided into three groups according to their distribution routes. Even though these studies have identified genetic differences across germplasm from various geographical origins, the underlying selection signatures are still not fully understood. This is mainly due to the large and complex genome of faba bean (approx. 13 Gbp) [5, 20]. Most previous studies did not study in depth the genetic diversity indices such as the number of different alleles, the number of common alleles, the number of private alleles, the expected heterozygosity, and unbiased heterozygosity. Understanding these genetic diversity indices provides an intellectual basis to select parents in the next faba bean breeding programs. Additionally, by using association analysis to find superior alleles that regulate the excellent traits of faba bean germplasm resources, this can also serve as a theoretical basis for the next step, which will support the use of molecular marker-assisted selection technology in the breeding of new faba bean varieties.

The objective of this study is to (1) characterize the allelic pattern, genetic diversity, Linkage disequilibrium, and population structure of the Egyptian faba bean and (2) investigate the potential effects of plant breeding and selection on genetic diversity and population structure.

## Material and methods

### Plant material

The plant material consisted of 128 faba bean genotypes which are originally from Egypt. These genotypes were collected from different sources namely,

1. Egyptian universities and research institutes (EURI): 15 genotypes were collected from different breeding programs established at some Egyptian universities and research institutes.
2. Leibniz Institute of Plant Genetics and Crop Research (IPK): a set of 102 faba bean genotypes was collected from the Department of Gene Bank, IPK (51°49′25.0"N 11°17′34.8"E), Germany.
3. Collaborative research institutes (CRI): SNP sequence data of the 11 faba bean breeding lines were selected from a previous study of [5]. The 11 genotypes were bred in the Andalusian Institute of Agricultural and Fisheries Research and Training (IFAPA, Spain; nine genotypes) (37°51′36.2"N 4°47′54.3"W), the International Centre of Agricultural Research in the Dry Areas (ICARDA, Syria; two genotypes) (34°27′08.5"N 6°51′37.7"W), and the French National Institute for Agriculture, Food and Environment (INRAe, France; one genotype) (50°38′28.7"N 3°08′31.0"E). The 11 genotypes belonged to the Europe and chinse legumes projects (EUCLEG) which was made in collaboration with public institutes [5].

The list of genotypes used in this study is presented in Supplementary Table 1. The EURI and IPK groups were self-propagated for three years in isolated cages at the Experimental Field Station, Department of Genetics, Assiut University.

### DNA extraction and genotyping

Initially, Genomic DNA was extracted from a set of 123 genotypes from the EURI and IPK group by TraitGenetics, Gatersleben, Germany. Then, all genotypes were genotyped using the Specific Primer Enrichment Technology (SPET) [21]. The results of genotyping revealed ~ 700K SNPs. The data were filtered to remove missing data of 30% and a minor allele frequency of 0.05. Finally, 31,250 SNPs on a total of 117 genotypes remained after marker and genotype filtration.

To include as many as faba bean genotypes in the panel of this study, the public sequence data published (21, 345 high-quality SNP markers) by [5] were exploited as they genotyped a large faba bean panel of 2,682 faba bean accessions. Out of the 2,682 faba bean genotypes, 11 were originally from Egypt. Fortunately, the 21, 345 SNP markers were generated by the SPET. The position of the 21, 345 SNP markers were compared to those positions of SNPs obtained in this study. As a result, 14950 SNP positions were matched and filtered using TASSEL5.0 [22] to produce a final set of 6,759 markers on 128 faba bean genotypes which were used for investing genetic diversity among the Egyptian faba bean in this study.

### Genetic properties of SPET SNP markers

The summary statistics of all 6759 SNP markers such as gene diversity(GD), polymorphism information content (PIC), minor allele frequency (MAF), and observed heterozygosity were calculated using PowerMarker software V 3.25 [23]. The PIC was calculated according to [24] as follow;

$${\text{PIC}}=1- \sum_{j=1}^{n}{P}_{ij}^{2} -\sum_{j=1}^{n=1}\sum_{k=j+1}^{n}{2P}_{ij}^{2}{P}_{ik}^{2}$$

where P_ij_ and P_ik_ are the frequencies of jth and k_th_ alleles for marker i, respectively.

### Analysis of population structure

To detect the correct number of subpopulations explaining population structure, Structure 2.2.3 was used as a clustering program [25]. The analysis was run with burn-in periods of 100,000 and 100,000 MCMC (Markov Chain Monte Carlo) replications and the ad hoc statistic [26] was used to determine the correct estimated number of clusters with Structure Harvester online program [27]. Subpopulation number (K) was tested from 2 to 10 with 20 iterations for each group. Subpopulations were determined according to a threshold of ≥ 0.70 inferred ancestries. Genotypes with an identity value under this threshold were considered to be intermixed.

### The analysis of molecular variance (AMOVA)

The analysis of molecular variance was performed using 6,759 SNP markers using GenAlex 6.41 software [28]. The AMOVA was performed among subpopulations resulting from STRUCTURE and the three groups (EURI, IPK, and CRI).

The alleleic pattern and genetic indices such number of loci with private alleles, number of different alleles (Na), number of effective alleles (Ne), diversity index (h), unbiased diversity (uh), and Shannon’s Information Index (I) were calculated using GeneAlEx 6.41 [28].

### Analysis of Linkage disequilibrium (LD)

The analysis of linkage disequilibrium (*r*^*2*^) of each pair of the 6759 SNPs was calculated using TASSEL v.5.2.5 software [22]. Moreover, for each chromosome, the LD was also calculated to understand the structure of LD in the current population. The significant LD between each marker pair was determined by Bonferroni correction at a significant level of 0.01. The LD decay for each genome was calculated according to [29] using R Software.

For each chromosome, the number of haplotype blocks was determined using Haploview 4.2 software [30]. To perform this, the SNP data on each chromosome was used to calculate the pair-wise LD between each SNP pair located on the same chromosome. The haplotype blocks were identified using the four-gamete method with a cutoff of 1% [31, 32].

## Results

### SNP markers distribution and their genetic properties across faba bean genome

**Fig. 1:**
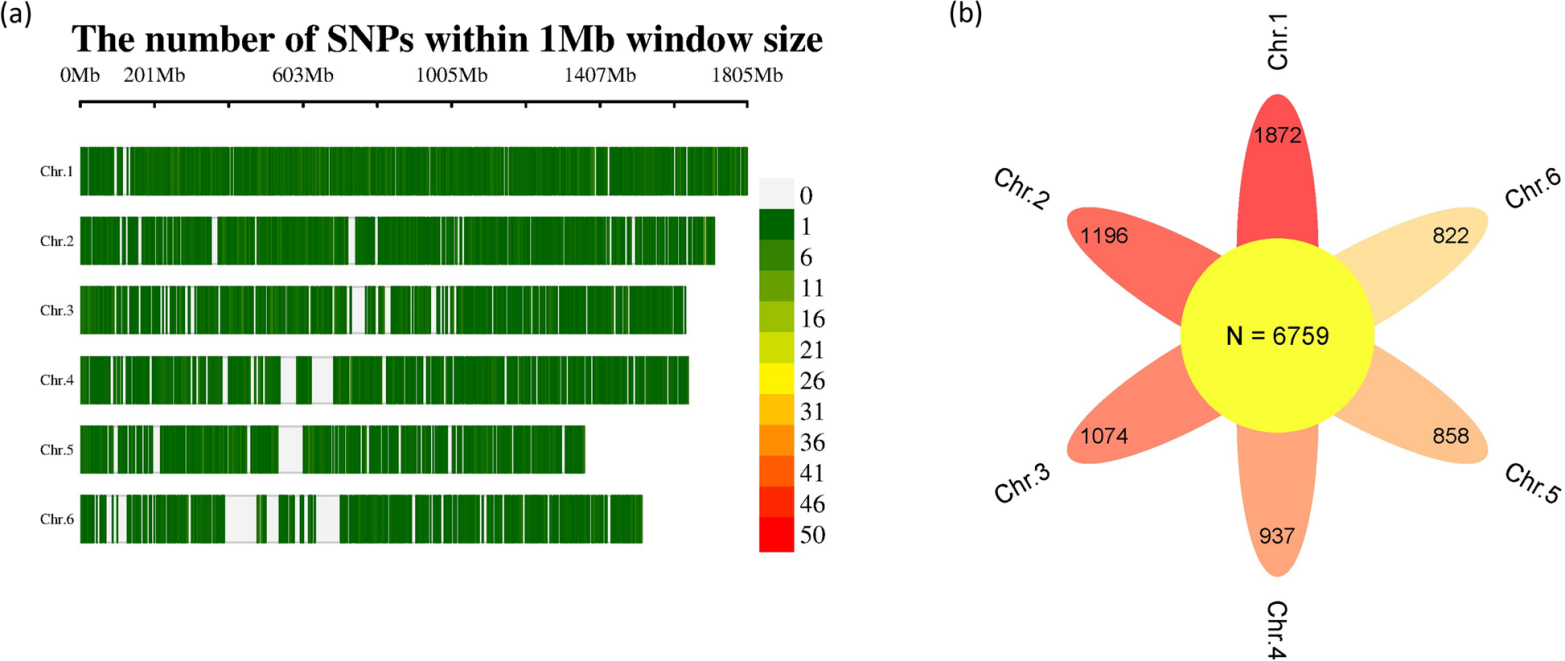
Distribution of SNP markers on faba bean chromosomes (**a**), total number of SNP markers on each chromosome (**b**)

**Fig. 2:**
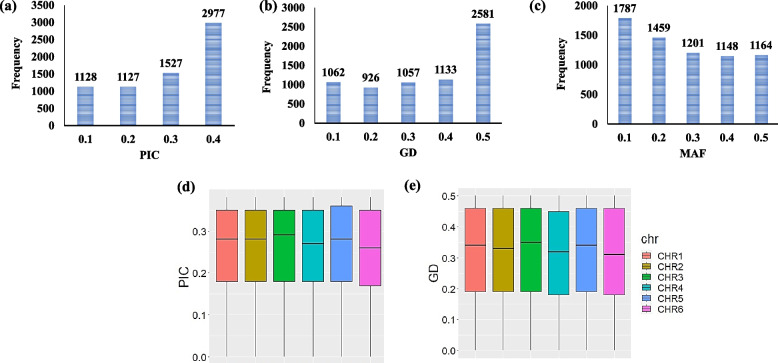
Genetic properties for SNP markers used in this study; the polymorphic information content (PIC) (**a**), the gene diversity (GD) (**b**), the Minor Allele Frequency (MAF), (**c**),  boxplot of PIC values on each chromosome (**d**), boxplot of GD values on each chromosome (**e**)

The SNP array revealed a set of 6,759 SNP markers that were distributed across the six faba bean chromosomes (Fig. 1a). The highest number of SNPs (1,872 markers) were found for chromosome 1, while chromosome 6 had the lowest number of SNP markers (822 markers) (Fig. 1b). The genetic properties of the SNP markers are presented in Fig. 2. The PIC values of the SNP markers ranged from 0.1 (1,128 markers) to 0.4 (2,977 markers) (Fig. 2a). Gene diversity analysis of the markers extended from 0.1 (1,062 markers) to 0.5 (2,581 markers) (Fig. 2b). Most of the SNP markers had a minor allele frequency of 0.1 (1,787 marks) (Fig. 2c). At the chromosomal level, the PIC and GD values for all markers located on each chromosome is presented in Fig. 2d and e, respectively. On average, markers in Chr. 3 had the highest average of PIC and GD, while, Chr. 6 had markers with the lowest average of PIC and GD. For PIC, Chr. 1, 2, and 5 had approximately the same average. The average of GD for all markers varies by chromosome.

### Population structure and relationships

**Fig. 3:**
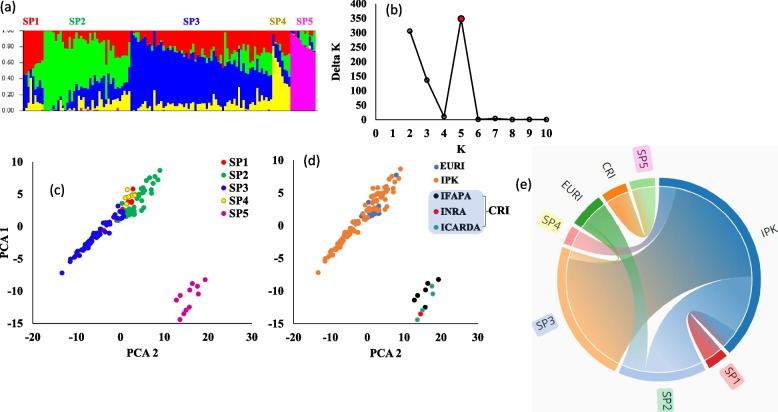
The analysis of population structure; subpopulations results from STRUCTURE software (**a**), Delta K is shown for various number of clusters (k) – the red point refers to the best number of K (**b**),  PCA based on the results of STRUCTURE (**c**),  PCA based of the groups (IPK, EURI, and CRI) (**d**), Distribution of the faba bean groups on the five subpopulations (**e**)

The analysis of STRUCTURE was performed on the 128 faba bean genotypes to explore the number of subpopulations (Fig. 3a). The number of K (clusters) was plotted against ΔK to find out the possible number of subpopulations in the Egyptian population. The largest ΔK value was observed at K = 5, indicating the presence of possible five subpopulations in the Egyptian faba bean genotypes (Fig. 3a and b). The five groups consisted of nine, 38, 62, eight, and 11 genotypes for the SP1, SP2, SP3, SP4, and SP5, respectively (Fig. 3a and Table 1). By comparing the results of the STRUCTURE software and the PCA, we found that both agreed and divided the tested genotypes into clear five groups (Fig. 3c). To determine the overall genetic variation among the subpopulations, the fixation index (Fst) was calculated by STRUCTURE (Table 1). Subpopulation 2 had the highest genetic variation with a Fst of 0.548, while, SP 4 showed the lowest genetic variation (Fs *t* = 0.067). The average distance (expected heterozygosity) among genotypes in each subpopulation is presented in Table 1. The expected heterozygosity ranged from 0.167 (SP2) to 0.341 (SP4). The Fst (fixation index) and Nm (gene flow) among the five subpopulations are presented in supplementary Table 2.

**Table 1:** Fixation index (Fst) (significant divergences), average distance (expected heterozygosity) and number of genotypes in each subpopulation resulted from STRUCTURE analysis of the 128 Egyptian faba bean genotypes

Subpopulation	Fst^a^	Exp. Hetero^b^	No. of genotypes
Subpopulation 1 (SP1)	0.372	0.246	9
Subpopulation 2 (SP2)	0.543	0.167	38
Subpopulation 3 (SP3)	0.288	0.279	62
Subpopulation 4 (SP4)	0.072	0.341	8
Subpopulation 5 (SP5)	0.239	0.310	11

^a^Fst is a measure of genetic differentiation; ^b^Average distances (expected heterozygosity) between individuals in the same cluster

Interestingly, the PCA was also performed to identify group patterns based on the breeding institute/university (Fig. 3d). Faba bean genotypes bred by ICARDA, INRAe, and IFAPA were separated from other faba bean genotypes bred at IPK and EURI. It was also noted that, all genotypes from IPK (102 genotypes) were distributed on SP1 (8%), SP2 (26%), SP3 (57%), and SP4 (9%), while the 15 faba bean genotypes from EURI were assigned to SP2 (73%) and SP3 (27%). All genotypes bred in IFAPA, INRAe, and ICARDA were assigned to SP5 (Fig. 3e), while the other genotypes were bred in Egyptian universities and research institutes (EURI).

### Analysis of molecular variance (AMOVA) among subpopulations

The AMOVA was performed on the five subpopulations resulting from STRUCTURE. The AMOVA, Fst, and a haploid number of migrants (Nm) are presented in Table 2. The results of AMOVA revealed that 12% of the total variation was found among subpopulations, while 88% of the variation was accounted for within among individuals. The haploid Nm was 1.902 and Fst was 0.534.

**Table 2:** Analysis of molecular variance and the genetic differentiation among and within five subpopulations of 128 Egyptian faba bean using the 6759 SNP markers

Source	df	SS	MS	Est. Var	%
**Among Pops**	4	47422.988	11855.747	206.124	12%
**Within Pops**	124	385694.199	3135.725	1567.863	88%
**Total**	255	433117.188		1773.987	100%
**Fixation Index (Fst)**	0.534				
**Nm (Haploid)**	1.902				

*df* degrees of freedom, *SS* sum of squares, *MS* mean square deviations, *Est.Var*. estimated variance component; percentage of total variance (%) contributed by each component and significance of variance (*p*-value); ** *p* < 0.001

It was worth investigating the variation among the Egyptian faba bean based on the breeding institute where they were collected from. The Egyptian faba bean genotypes were mainly collected from two sources: the IPK (Germany) and EURI (Egypt). The remaining 11 genotypes were from IFAPA, INRAe, and ICARDA as they are collaborative research institutes (CRI) in legume breeding research. Because the STRUCTURE assigned these 11 genotypes to one subpopulation SP5, these genotypes were considered as one source. Therefore, all the Egyptian faba bean genotypes were divided into three groups IPK, EURI, and CRI. The AMOVA was performed among these groups and the results are presented in Table 3. The AMOVA revealed 19% of the total variation was found among subgroups, while the rest of the variation (81%) was among individuals. The haploid Nm was 1.032 and Fst was 0.538.

**Table 3:** Analysis of molecular variance and f the genetic differentiation among and within the three groups  (IPK, EURI, and CRI) using the 6759 SNP markers

Source	df	SS	MS	Est. Var	%
**Among Pops**	2	39834.121	19917.060	381.019	19%
**Within Pops**	126	393282.254	3146.258	1573.129	81%
**Total**	255	433116.375		1954.148	100%
Fixation Index (Fst)	0.538				
Nm (Haploid)	1.032				

*df* degrees of freedom, *SS* sum of squares, *MS* mean square deviations, *Est.Var*. estimated variance component; percentage of total variance (%) contributed by each component and significance of variance (*p*-value); ** *p* < 0.001

### Genetic distance among the Egyptian faba bean genotypes

**Fig. 4:**
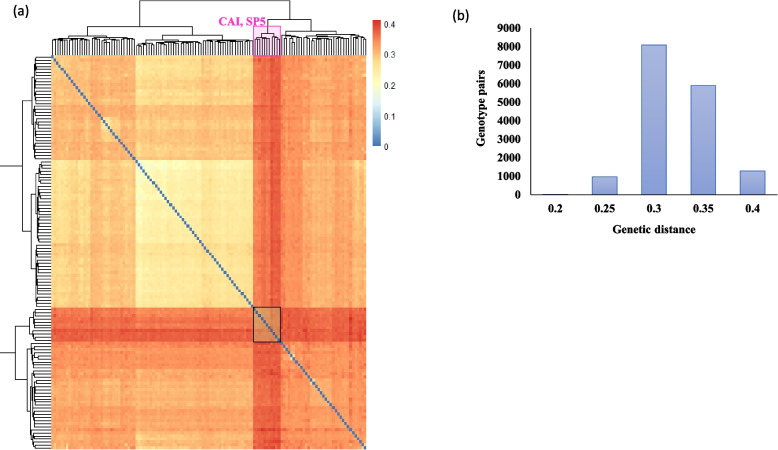
Heatmap showing genetic distance among the Egyptian faba bean (**a**), Number of genotype pairs in each genetic distance class (**b**)

The genetic distance among the 128 Egyptian faba bean genotypes was calculated to study the highly divergent genotype pairs (Fig. 4a, supplementary Table 3). The distribution of genetic distance between each genotype pair is presented in Fig. 4b. The genetic distance ranged between 0.1 (FB-227 and FB-232) to 0.6 (FB-028 and EUC_VF_194) The majority of genotypes had a genetic distance between 0.4–0.45. Interestingly, EUC_VF_194, EUC_VF_220, NS315, NS316, EUC_VF_336, and EUC_VF_337 had high genetic distance (0.46–0.61) with all genotypes.

### Allelic pattern across the populations

The mean value of different alleles (Na), number of effective alleles (Ne), Shannon’s index (I), the diversity index (h), and the unbiased diversity index (uh) for each subpopulation resulting from STRUCTURE are presented in Table 4. SP4 had the lowest average in Na, Ne, I, h, and uh. On the other hand, SP5 had the highest value in allele pattern and diversity indices.

**Table 4:** “Mean of different genetic parameters including number of different alleles (Na), number of effective allele (Ne), Shannon’s index (I), No. of common allele, diversity index (h), unbiased diversity index (uh), and in each subpopulation of the 128 Egyptian Faba bean

Population	Na	Ne	I	He	uHe
*STRUCTURE*					
SP 1	1.592	1.369	0.324	0.217	0.235
SP 2	1.789	1.380	0.359	0.232	0.239
SP 3	1.744	1.298	0.291	0.185	0.187
SP 4	1.325	1.225	0.192	0.131	0.150
SP 5	1.717	1.413	0.373	0.247	0.262
*Group (Breeding institute/university)*					
IPK	1.870	1.354	0.341	0.217	0.219
EURI	1.464	1.313	0.269	0.183	0.207
CRI	1.717	1.413	0.373	0.247	0.262

Na = No. of Different Alleles with a Frequency >  = 5%

Ne = No. of Effective Alleles = 1 / (Sum pi^2)

I = Shannon's Information Index = -1* Sum (pi * Ln (pi))

No. Private Alleles = No. of Alleles Unique to a Single Population

No. LComm Alleles (< = 50%) = No. of Locally Common Alleles (Freq. >  = 5%) Found in 50% or Fewer Populations

He = Expected Heterozygosity = 1—Sum pi^2

uHe = Unbiased Expected Heterozygosity = (2N / (2N-1)) * He

Looking at the allelic patterns and diversity indices among the three groups, classified based on breeding institutes, the CRI group had the highest allelic pattern and diversity except for Na which was the highest for the IPK group. The EURI group had the lowest allelic pattern and diversity indices.

**Fig. 5:**
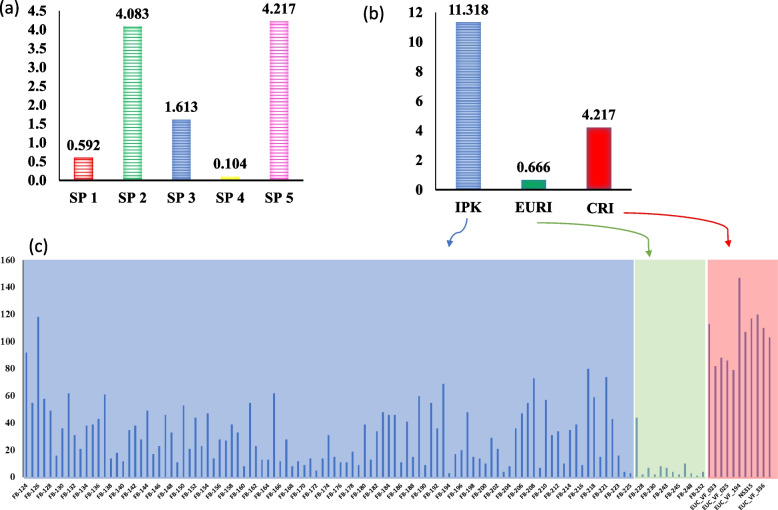
No. of private alleles in each subpopulation (**a**),  no. of private alleles in each group (**b**), no. of private alleles in each genotype (**c**)

The proportion of private alleles in each subpopulation is illustrated in Fig. 4a. SP5 had the highest proportion of private alleles (4.2%) followed by SP2 (4.1%), while the lowest proportion of private alleles was found for SP4 with 0.1% (Fig. 5a). According to the classification based on the breeding institutes, IPK had the highest proportion of private alleles (11.3%) followed by CRI (4.2%), and EURI (0.6%) (Fig. 5b). By looking at the private allele in each genotype, it was noted that the 11 genotypes (CRI group) assigned to SP5 had the highest number of private alleles compared to all other genotypes (Fig. 5c). Among the 11 genotypes, EUC_VF_023 had 82 alleles, while, EUC_VF_194 had 147 private alleles (Fig. 5c).

### Linkage disequilibrium structure in faba bean genome

**Fig. 6:**
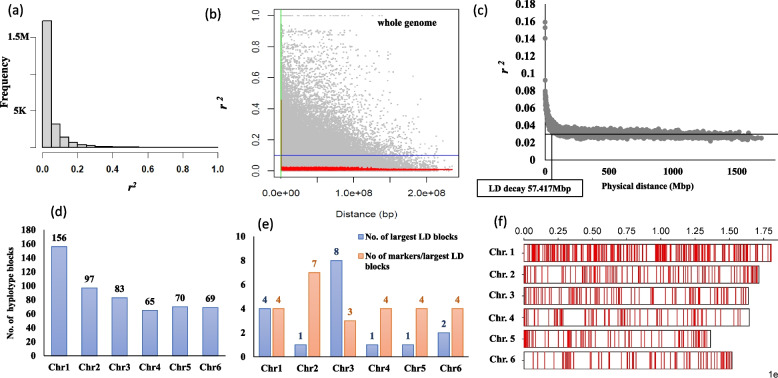
The frequency of LD pattern among each SNP pair across faba bean genome (**a**), LD decay across faba bean genome using individual marker pair (**b**), LD decay across faba bean illustrated by plotting the average of LD and distance in bins of 1000 SNP pairs (**c**), no. of haplotype block on each chromosome (**d**), no. of largest LD blocks (having high marker density), the number of marker in the largest block (**e**), and the distribution of haplotype blocks on each faba bean chromosome

The linkage disequilibrium structure (*r*^2^) was investigated in the faba bean genome. The frequency of LD (*r*^*2*^) between each pair of SNPs is illustrated in Fig. 6a. The majority of SNP pairs across faba bean genome had an r^2^ of =  > 0.4. The analysis of the LD structure on each chromosome is presented in Table 5. The mean value of *r*^*2*^ was very low in each chromosome. Chromosome 1 had the highest number of marker pairs that had high significant LD with *r*^*2*^ of 0.5823. The average of *r*^2^ for the significant marker pairs was approximately the same in chromosomes 3, 4, 5, and 6 (*r*^*2*^ ~ 0.48). The majority of marker pairs that had non-significant LD had r^2^ extending from 0.0047 (Chr. 1) to 0.0085 (Chr. 2).

**Table 5:** Linkage disequilibrium between SNP marker pairs located on the same chromosome in faba bean

Chr	*r*^*2*^ mean	No. of sig. LD	Average of sig. LD	No. of non-sig LD	Average of non-sig. LD
Chr.1	0.0050	619	0.5823	1654607	0.0047
Chr.2	0.0082	225	0.5169	685336	0.0082
Chr.3	0.0055	232	0.4893	549520	0.0053
Chr.4	0.0058	185	0.4926	410888	0.0056
Chr.5	0.0054	117	0.4639	348265	0.0052
Chr.6	0.0059	141	0.4992	312700	0.0059

No. of sig. LD: number of marker pirs that were significant in LD at 0.01 Bonferroni correction

Average of sig. average of *r*^*2*^ (LD) for significant markers

No. of non-sig LD: number of marker pairs that were non-significant in LD

Average of sig. average of *r*^*2*^ (LD) for nonsignificant markers

**Fig. 7:**
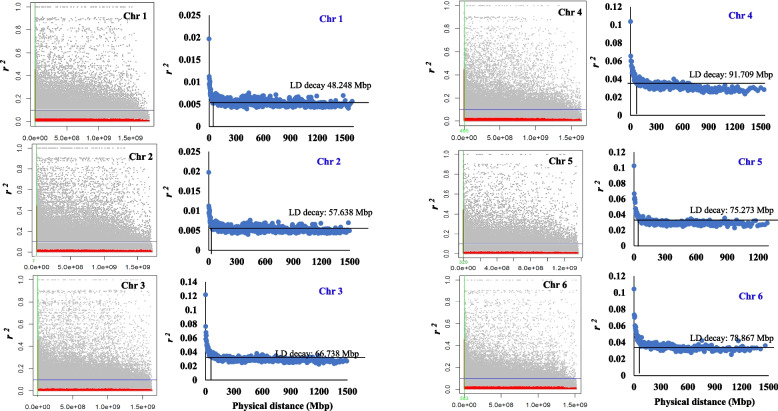
LD decay on each faba bean chromosome using individual marker pair,  LD decay on each faba bean chromosome illustrated by plotting the average of LD and distance in bins of 1000 SNP pairs

The LD between each pair of SNPs was plotted against the genetic distance (pb) to identify the LD decay (Fig. 6b). To better visualize the LD decay, the physical distance (Mbp) between marker pairs was binned into groups of 1000 after sorting and the average was calculated. Then the average of the physical distance was plotted again the corresponding average of r^*2*^ (Fig. 6c). The LD decayed and dropped at 57.417Mbp across the faba bean genome. Moreover, the number of significant haplotype LD blocks on each chromosome is illustrated in Fig. 6d. Chromosome 1 had the highest number of significant LD blocks with 156 blocks, while a total of 65 significant LD blocks were found in Chr.4. The number of LD blocks included SNPs ranged from two to seven markers. Notably, Chromosome 3 had the highest number of largest LD blocks with three markers each, while only one large block with eight markers in that block. To investigate the number of markers in each haplotype block, we determined the number of largest high LD blocks (Fig. 6e). Chromosomes 2, 4, and 5 had one LD block that included seven, four, and four SNP markers, respectively. Chromosome 3 had eight large LD blocks with three SNPs in each. The distribution of the haplotype blocks across each chromosome is presented in Fig. 6f. At the chromosomal level, the LD was also decayed at 48.248 Mbp in chromosome Chr. 1, while it was dropped in Chr. 6 at 78.867 Mbp (Fig. 7).

## Discussion

Due to the high protein content in faba bean crop, it has been a pole of the Egyptian diet. Although it is considered the first leguminous food crop in Egypt, very few faba bean cultivars (~ 10–15 faba bean cultivars) are widely grown in Egypt. This small number of highly adapted faba bean genotypes may be vulnerable to extinction due to the consequences of climate change. Therefore, expanding the circle of genetic diversity in faba bean genotypes especially those with high adaptiveness to the Egyptian conditions should be highly considered. Therefore, faba bean genotypes were collected from different research institutes/universities to investigate the level of genetic diversity among faba bean genotypes which were originally collected from Egypt. As a result, a set of 102 faba bean genotypes were found at the Leibniz Institute of Plant Genetics and Crop Plant Research (Germany), nine of IFAPA (Selvia), and two from ICARDA (Syria). All these genotypes in addition to a total of 11 widely-grown Egyptian faba bean were used in this study to genetically dissect the genetic diversity of an Egyptian fava bean panel.

Understanding the genetic diversity of a target crop panel is an essential step for successful breeding programs to genetically improve important traits such as grain yield and tolerance to various biotic and abiotic stresses. For the past decades, the complicity of the faba bean genome hindered the dissection of genetic diversity in this important legume crop. Therefore, few research efforts have been made to explore genetic diversity in faba bean, compared to other crops, with a limited number of DNA markers [14, 33–36]. The utilization of transcriptome analysis has also led to the development of a high-density faba bean genotyping array named SPET genotyping which includes ~ 60 K probes [37]. The SPET method was previously used to genotype 2,678 faba bean genotypes and it generated 21,345 SNP markers [5]. In this study, a set of 6,759 SNPs resulted from the genotyping by SPET method and was used to dissect the genetic diversity of the Egyptian panel used in this study.

The 6, 759 SNPs were distributed across all faba bean chromosomes, indicating the usefulness of these markers for detecting as many marker-trait associations in this population. The PIC values of these SNP ranged from 0.04 to 0.375 which was approximately a similar range reported by [19] (0.09–0.375) and [11] (0.05–0.38). Compared to other markers (e.g. SSR), the highest PIC value for bi-allelic markers (e.g. SNPs) is 0.5. Therefore, SNP markers could be classified as moderate to low informative markers. However, the recent sequencing methods generate a lot of SNP markers that can be utilized to deeply study the genetic diversity among genotypes [37] reported that PIC values indicate the informativeness of SNP markers which could be utilized in investigating the genetic diversity in different organisms [38, 39]. The range of gene diversity of the markers in this study was also similar to those reported in earlier studies that used different population sizes and different numbers of SNPs. Moreover, the highly informative markers were distributed across all faba bean chromosomes, allowing the detection of target SNPs for important traits to be feasible. The distribution of informative markers reflects the overall diversity in the crop populations [39, 40]. The PIC values and gene diversity are very useful in assessing the polymorphism among the genotypes for breeding and genetic diversity programs [39, 41]. Based on the distribution of PIC and gene diversity values in our tested faba bean population, we can conclude that these markers explained the genetic diversity among the Egyptian faba bean population and could be used in other genetic studies such as genome-wide association studies to identify alleles controlling target traits.

The majority of SNPs had a minor allele frequency of ~ 0.1 and they were distributed across the genome. The distributions of allele frequencies shed light on the population genetic architecture of complex traits [42]. The lower allele-frequency SNPs will certainly help in discovering new loci and provide a further investigation of different population genetic models for further understanding of differences and similarities among genotypes [42].

The genetic properties of the SNP markers used in this study on the Egyptian faba bean populations indicated the efficiency of the SPET method in generating informative SNPs that can be utilized in genetic studies in faba bean.

### Population structure and relationship

The analysis of population structure (PS) provides important information on the level of genetic diversity among the tested genotypes, and it is one of the basic analyses that should be conducted before performing genome-wide mapping studies. Among the clustering methods, the STRUCTURE software used in this study is the most recommended analysis to explore the possible subgroups in the tested population [43]. The STRUCTURE analysis divided the Egyptian faba bean population into possible five subgroups (SP1, SP2, SP3, SP4, and SP5). The PCA analysis was in agreement with the STRUCTURE results. The presence of five subgroups in the current population was surprising as all genotypes were originally from Egypt. The cross-pollination nature of the faba bean crop and the effect of the environment may explain the structure found in the current population. Most of the faba bean genotypes (102 genotypes) were collected from IPK (Germany) in which this material was grown for many years in an open-pollination field. Also, a set of 11 faba bean genotypes were bred in IFAPA (Spain), INRAe (Hungary), and ICARDA (Syria). Therefore, it is expected that new genes were integrated into, by pollinators, these materials by the gene flow due to the immigration (seed exchange) of these genotypes from out of the origin (Egypt). The IPK group genotypes were distributed on four subpopulations (SP1, SP2, SP3, and SP4). The EURI group bred in Egypt shared the IPK group in SP2 and SP3. The PCA revealed that the EURI and IPK were very near to each other. The Nei genetic distance of 0.060 further supported the closeness of these two groups (IPK and EURI), while it was 0.135 and 0.164 between CRI and IPK and between CRI and EURI, respectively (supplementary Table 4). Remarkably, the CRI group (ICARDA, IFAPA, and INRAe) was clearly separated from the IPK and EURI groups creating a distinct subgroup (SP5). The distinct separation between CRI and the other two groups may be due to the fact that the genotypes collected from CRI were exposed to strong gene flow before breeding these lines by single seed descent. The Nm (gene flow) value was less than 1 between EURI and IPK (0.89) and between EURI and CRI (0.46) (Supplementary Table 2). When Nm < 1, this indicates that populations have different genetic structures that may be due to the evolutionary change through the adaptation to the local environments via natural selection or through genetic drift [44]. Bearing in mind that the IPK and CRI were bred and collected from European countries (Germany, Sapin, and Hungary). The Nm between CRI and IPK was 1.8, indicating considerable variation in gene frequencies among these two population populations [45]. Therefore, the gene flow could be one of the main reasons for this clear separation of CRI from the other two groups (IPK and EURI). The IPK genotypes were distributed in the two subpopulations. Among the 15 EURI genotypes, 14 were assigned to subpopulation 2 and only one was assigned to subpopulation 1. The structure analysis was performed on the 128 genotypes (Fig. 3e), EURI 14 genotypes were assigned to SP2 where all other members were from the IPK and only one genotype was assigned to SP3 where the other members were from the IPK group. Another reason for this clear separation of the CRI could be due to that these 11 genotypes had shared faba bean ancestor and after migration, it could be grown in an open field, then collected by the research institutes that bred this ancestor by single seed descent to get highly homozygous breeding lines. Interestingly, the CRI (SP5) genotypes were previously included among 2678 faba bean genotypes in a genetic diversity study reported by [5] who assigned the 11 genotypes in the same subpopulation (SP2), confirming the results of structure analysis performed in this study. Although STRUCTURE revealed the possible genetic subpopulations, however, the analysis of genetic features based on the breeding institute (IPK, EURI, and CRI) was very useful in unlocking the genetic diversity and population structure among these genotypes.

The analysis of AMOVA revealed a high percentage of variation among subpopulations (12%) and groups (19%) which was higher than was reported by Zhang et al. [19] who found only 1% among subpopulations from a set of 410 global faba bean accession. This indicates the presence of high diversity among Egyptian faba bean genotypes.

### Genetic distance among the genotypes

The genetic distance found among genotypes further indicates the existence of considerable genetic diversity among genotypes. The heatmap of genetic distance revealed high similarity among the 11 genotypes and higher genetic distance with all genotypes. Therefore, crossing among genotypes will be fruitful for improving target traits such as seed yield and tolerance to biotic and abiotic stress tolerance. The EUC_VF_194 had a high genetic distance with all genotypes, therefore these genotypes may be very useful to be integrated into future breeding programs. Crossing among highly divergent genotypes will lead to producing cultivars having high-yield traits [41, 46]. Fortunately, the IPK groups were tested for two growing seasons under the Egyptian conditions for their yield traits and they are highly adapted under the Egyptian conditions (unpublished data – Ahmed Sallam, personal communication). Moreover, the same population was phenotyped under severe drought stress in 2022/2023 [47]. High significant genetic variation was found among genotypes in plant height (32.7 – 86.95 cm), stay green (1.5–9), day to flowering (31.18—62 days), and days to maturing (124.5 – 146.22 days). Therefore, the genetic improvement of yield traits under various biotic and abiotic stress is feasible are feasible in this population. As previously mentioned, there are ~ 15 known faba bean genotypes in Egypt that are widely grown, this very low number of genotypes could threaten the food security of Egypt for this important legume. Therefore, integrating the genotypes for the IPK and CRI into Egypt will not only improve the production and productivity of faba bean in Egypt but also it will expand the circle of genetic diversity of this important crop to face the dangerous challenges of climate change.

### The allelic pattern among the subpopulation

The allelic pattern among the subpopulations indicated that SP5 (CRI) had the highest values of all the indices. So, the SP5 subpopulation could provide a good source of genetic diversity in faba bean. As previously discussed, including genotypes from SP5 (CRI) will be a very future faba bean breeding program after evaluation for the target traits—private alleles. More importantly, private alleles either among subpopulations or among genotypes shed light on a remarkable differentiation in the loci. Estimating private alleles provides important information on those presented in only subpopulations. In this regard and as was expected, genotypes from SP5 (CRI) had the highest number of private alleles among other subpopulations. Again, the EUC_VF_194 genotype had the highest number of private alleles ( PA < 140), distinguishing this genotype from the other genotypes in the population. The private alleles highlight the unique genetic variability in certain loci and the identification of highly diverse genotypes that could be utilized in crop breeding programs as candidate parents to maximize the allele richness in the population [48]. The EURI had the lowest percentage of private alleles compared to CRI and IPK. Therefore, it is probable that the genotypes in CRI and IPK might acquire new loci and genes, before and during breeding research, that did not exist in the EURI.

### The structure and extent of LD in the genome of the Egyptian faba bean population

Understanding the LD magnitude and the decay are essential for obtaining high mapping resolution in genome association analyses because they provide an idea of the number of SNP markers required for performing association analyses [49]. At the genomic level, LD extent differs by species [49]. The characters of haplotype blocks in faba bean genome in the current population were studied and they varied across all chromosomes. The haplotype blocks in the genome shed light on the important genomic regions of interest that may include candidate genes or studying different genotype groups at specific loci [50]. Interestingly, the haplotype blocks were distributed across each chromosome indicating the possibility to identify candidate genes for target traits in this population.

Most of the marker pairs were found to have *r*^*2*^ of between 0–0.1, indicating the presence of very low LD in the genome of the Egyptian faba bean. Interestingly, in the whole genome and each chromosome, high LD genomic regions (between marker pairs) were observed at high genetic distances (Fig. 7) when the *r*^*2*^ between each marker was plotted against the phsyical distance. These high LD genomic regions which were among low LD (non-significant) regions can be considered LD hotspots. Therefore, it is essential to identify the structure of LD in the faba bean population and the distribution of LD hotspot regions across the genome. In the current population, the LD decayed below *r*^*2*^ of 0.1 at a drop point of 57.417 Mbp which at a lower physical distance than reported by Skovbjerg et al. [5] in seven-parent MAGIC (LD decay =  ~ 68 Mbp) and four-way cross (LD decay =  ~ 77 Mbp) faba bean populations. The LD decay in some other faba bean populations was found to be faster e.g. at 681, 730 bp in EUCLEG, 678,648 bp in NORFAB, and 672,877 bp in ProFaba [5]. This difference in LD decay at different physical distances is due to the number of recombinations in each faba bean population. Therefore, it is very important to measure the LD decay in the faba bean population, especially in genetic association studies. Genetic diversity has a definite relationship with linkage disequilibrium (LD) decay, which successively affects the diversity and LD-based association mapping [51].

It is worth investigating the LD decay on each chromosome for further understanding of the extent of LD in the Egyptians. Compared to the LD decay across the genome (Fig. 6c), the LD decay dropped very fast in Chr.1 at 42.248 Mbp, while it was at a high physical distance of 91.709 Mbp in Chr.4. This can be interpreted by observing the number of significant LD blocks which was the highest in Chr. 1 and lowest in chromosome 4. Selection, genetic drift, migration, and the nature of pollination (partially allogamous) could be strong reasons for this rapid decay and low LD in the Egyptian faba bean.

In conclusion, the detailed genetic diversity and population structure analyses performed on the Egyptian faba bean promised the genetic improvement of faba bean crop under Egyptian conditions. Selection of the promising genotypes as candidate parents in this panel is feasible as considerable genetic distance among genotypes was found. The LD structure performed on this process will be helpful in the genetic association study to identify candidate genes associated with target traits in Faba bean (e.g. grain yield, protein content, and tolerance to various biotic and abiotic stress tolerance).

### Supplementary Information

**Supplementary Material 1. **

## Data Availability

The sequence data that support the findings of this study are available from the authors, but restrictions apply to the availability of these data, which were used under license from the Academy of Scientific Research and Technology (ASRT) and Assiut University, Egypt for the current study, and so are not publicly available. Data are, however, available from the authors upon reasonable request and with permission from the Academy of Scientific Research and Technology (ASRT) and Assiut University, Egypt. All other data generated or analyzed during this study are included in this published article and its Supplementary information files.
