# Supplementary figures and images for: Molecular genetic diversity and linkage disequilibrium structure of the Egyptian faba bean using Single Primer Enrichment Technology (SPET)
Source: BMC Genomics. 2024 Jun 28;25:644. doi: 10.1186/s12864-024-10245-x (PMC11212244; doi:10.1186/s12864-024-10245-x)

## Slide 1
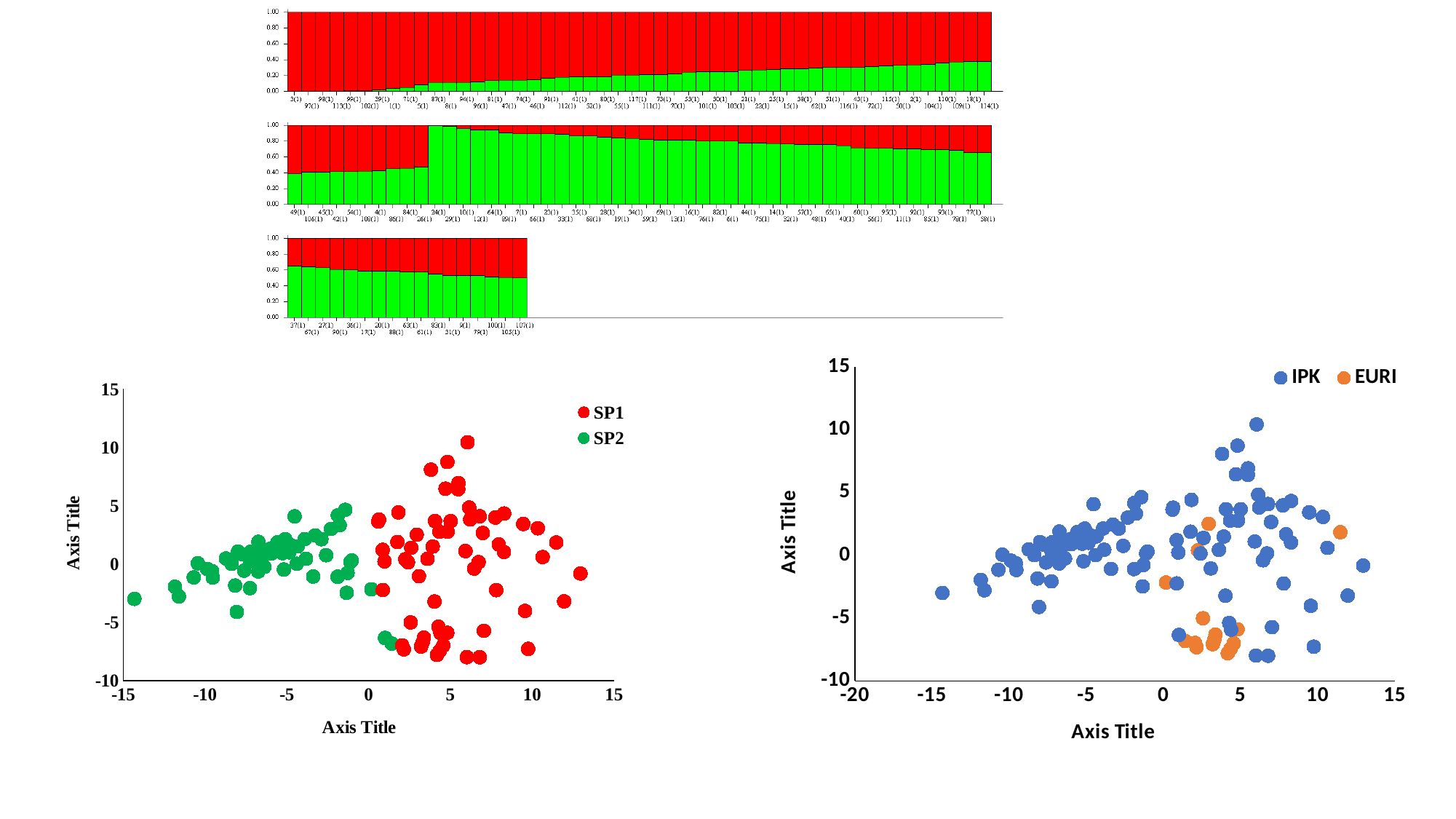

### Chart
| Category | | |
|---|---|---|
### Chart
| Category | | |
|---|---|---|

Supplement: Supplementary file 1 — Supplementary Material 1. [file 12864_2024_10245_MOESM1_ESM.zip › supp figures.pptx]
